# Frequency and Outcomes of Patients Presenting with Non-ST Elevation Myocardial Infarction (NSTEMI) without Standard Modifiable Risk Factors: A US Healthcare Experience

**DOI:** 10.3390/jcm12093263

**Published:** 2023-05-03

**Authors:** Jeffrey L. Anderson, Stacey Knight, Heidi T. May, Viet T. Le, Tami L. Bair, Kirk U. Knowlton, Joseph B. Muhlestein

**Affiliations:** 1 Intermountain Medical Center Heart Institute, 5171 So. Cottonwood Street, Building 1, 5th Floor, Murray, UT 84107, USA; stacey.knight@imail.org (S.K.); heidi.may@imail.org (H.T.M.); viet.le@imail.org (V.T.L.); tami.bair@imail.org (T.L.B.); kirk.knowlton@imail.org (K.U.K.);; 2 Department of Internal Medicine, The University of Utah School of Medicine, Salt Lake City, UT 84112, USA; 3 The Rocky Mountain University of Health Professions, Provo, UT 84606, USA

**Keywords:** myocardial infarction, non-ST-elevation, NSTEMI, risk factors

## Abstract

Patients with ST-elevation myocardial infarction (STEMI), but without standard modifiable risk factors (SMuRF-less), are surprisingly common and appear to have a worse, or at best similar, short-term prognosis. However, relatively little attention has been paid to the prevalence and prognosis of SMuRF-less patients with non-STEMI (NSTEMI). The aim of our study was to identify the proportion and outcomes of SMuRF-less NSTEMI patients in a large US healthcare population. Patients with NSTEMI between 2001–2021 presenting to Intermountain Healthcare hospitals and catheterization laboratories were included. SMuRF-less status was defined as no clinical diagnosis of, or treatment for, hypertension, hyperlipidemia, diabetes, and smoking. Outcomes were assessed at 60 days and long-term for major adverse cardiovascular events (MACE: death, myocardial infarction, and heart failure hospitalization). Multivariable Cox proportional hazard regression was used to determine MACE hazard ratios (HR) for SMuRF-less versus patients with SMuRF. NSTEMI patients totaled 8196, of which 1458 (17.8%) were SMuRF-less. SMuRF-less patients were younger, more frequently male, had fewer comorbidities, and were slightly less likely to have revascularization. For SMuRF-less patients, 60-day MACE outcomes were lower (adj HR = 0.55, *p* < 0.0001), and this persisted for long-term MACE outcomes (adj HR = 0.64, *p* < 0.0001) and for each of its components. In this large US healthcare population, SMuRF-less NSTEMI presentation, as with STEMI presentation, was found to be common (17.8%). However, unlike STEMI reports, short- and long-term outcomes were better for SMuRF-less patients. Further studies to increase understanding of risk factors and preventive measures for NSTEMI in SMuRF-less patients are indicated.

## 1. Introduction

Reports from outside of the United States recently identified and characterized a population of patients with ST-elevation myocardial infarction (STEMI) without standard modifiable risk factors, designated “SMuRF-less”, and have noted that they are surprisingly common (14–27% of STEMI cases) [1,2,3,4,5]. These studies have reported them to have a worse, or at best similar, short-term prognosis. We recently assessed the proportion and outcomes of SMuRF-less STEMI patients within our healthcare population. We confirmed their common occurrence (26%) among STEMI presentations. However, in contrast to some other earlier reports, the early prognosis was similar rather than worse than that of patients with SMuRF, and the long-term prognosis tended to be better [6]. 

There has been less focus on and understanding of MI patients presenting without ST-segment elevation, even though they are 2–3 times more common than STEMI [3,6,7,8]. Further, to our knowledge, there have been no reports of SMuRF-less NSTEMI patients’ prevalence and prognosis from United States healthcare systems. Therefore, the aim of the present study was to identify the proportion and short- and long-term outcomes of SMuRF-less NSTEMI patients in our large US healthcare population. 

## 2. Materials and Methods

### 2.1. Study Aims and IRB Approval

The primary study aim, which was a companion study to that of SMuRF-less STEMI patients [6], was to assess the prevalence of SMuRF-less patients presenting with NSTEMI to the emergency departments and catheterization laboratories of Intermountain Healthcare and to determine their cardiovascular prognosis compared to patients with standard modifiable risk factors (SMuRF). This database study, which was retrospective and observational in design, was approved with a waiver of consent by the Intermountain institutional review board (IRB).

### 2.2. Intermountain Health System and NSTEMI Pathways 

As noted in our earlier report [6] Intermountain Health is a nonprofit, integrated healthcare system that included up to 24 hospitals and 215 clinics in Utah, Idaho, and Nevada during the timeframe of this study. Intermountain has a longstanding (>25 years) centralized electronic medical records system and, in addition, a complementary database containing catheterization laboratory records. Intermountain has developed an acute coronary syndrome (ACS)-care pathway whereby ACS patients are efficiently triaged to a PCI-capable hospital. In-hospital coronary angiography is part of the general strategy for clinically significant NSTEMI presentations.

### 2.3. Study Population and Definitions 

The study population was comprised of patients with NSTEMI presenting to an Intermountain catheterization laboratory between 1 June 2001 and 31 January 2021. NSTEMI diagnosis at Intermountain includes a supportive clinical presentation together with an elevated (rising, falling) troponin level. Standard modifiable risk factors included a clinical diagnosis of, or treatment for, hypertension, hyperlipidemia, diabetes, and/or smoking (current or former). These diagnoses were determined from the Intermountain records database (by ICD-9 or -10 entry) and catheterization laboratory records. In our healthcare system, repeated systolic blood pressures ≥ 140 mmHg, total cholesterol ≥ 200 mg/dL, or hemoglobin A1C ≥ 6.5% are considered diagnostic of hypertension, hypercholesterolemia, and diabetes, respectively.

### 2.4. Study Endpoints

The primary short-term study endpoint was a major adverse cardiovascular event (MACE), which was defined as the occurrence of death (of any cause), myocardial infarction (MI), or a heart failure hospitalization (HFH) within 60 days of NSTEMI. The primary long-term MACE identified these outcomes up to the end of follow-up, i.e., until 3 March 2021. Secondary endpoints included individual event outcomes. 

### 2.5. Statistical Analysis

Analyses compared the occurrence of MACE in NSTEMI patients presenting with versus without standard modifiable risk factors, first during short-term (60-day) and then during long-term follow-up. Differences in baseline characteristics and medications were assessed using the chi-square statistic and the t-test as appropriate. Cox hazard regression analysis was used, both unadjusted and adjusted (adj). Analyses were adjusted for baseline patient and clinical factors that were significantly associated with SMuRF status or SMuRF risk factor from the chi-square test and t-tests. Stratified analyses by important baseline characteristics were made that compared both short- and long-term MACE outcomes.

## 3. Results

### 3.1. Patient Demographics

We identified 8196 qualifying NSTEMI patients. Of these, 1458 (17.8%) were SMuRF-less. Baseline characteristics of STEMI patients are presented in Table 1 and separated by SMuRF status. As with our STEMI cohort [6], SMuRF-less NSTEMI patients were younger, less often female, and were predominately White/Caucasian. SMuRF-less patients had fewer comorbidities other than the standard modifiable risk factors. These included lower frequencies of atrial fibrillation, COPD, depression, heart failure, stroke, and a family history of CVD (Table 1).

### 3.2. Angiographic Findings and NSTEMI Treatment

In SMuRF patients, coronary angiography was abnormal in 90.1% and normal in 9.9%. In SMuRF-less patients, angiography was abnormal in 83.6% and normal in 16.4% (*p* < −0.0001). NSTEMI treatment is summarized in Table 2. PCI was performed frequently in both groups but slightly less in SMuRF-less patients (52.6% vs. 58.1%, *p* = 0.0001). Similarly, coronary artery bypass surgery (CABG) was performed in a slightly smaller proportion of SMuRF-less patients (10.7% vs. 12.9%, *p* = 0.02). These differences are likely explained by the somewhat higher rate of normal or near-normal angiograms in SMuRF-less patients, which also may indicate an increased rate of MINOCA (myocardial infarction with no obstructive coronary artery disease) in SMuRF-less patients. Antiplatelet agents were prescribed to nearly all patients (>95%) in both groups. Beta-blockers were prescribed to a high and similar percentage (84%), and ACEIs or ARBs in a majority, but favoring SMuRF patients (62% vs. 51%, *p* < 0.0001). Statins were prescribed to ≥90% at discharge, slightly less in SMuRF-less patients, perhaps because of their absence of clinical hyperlipidemia, a higher rate of normal angiograms, or by chance.

### 3.3. Short-Term Outcomes

The short-term cardiovascular outcomes of NSTEMI patients are summarized in Table 3 by SMuRF status. There were a total of 590 early cardiovascular events (within 60 days), with rates of 4.05% in SMuRF-less patients and 7.88% in SMuRF patients (<0.0001). The adjusted hazard ratio (adj HR) of 0.55 (95% CI 0.42, 0.73; *p* < 0.0001) strongly favors SMuRF-less patients. All-cause death was the most frequent outcome (*n* = 452), and again, the adj HR strongly favored SMuRF-less patients (HR = 0.62 (0.46, 0.84); *p* = 0.002). There were fewer recurrent MIs (*p* = 0.02) and fewer heart failure hospitalizations in SMuRF-less patients (*p* = 0.03), although the numbers were too few to calculate adj HR.

### 3.4. One-Year and Long-Term Outcomes

**Figure 1:**
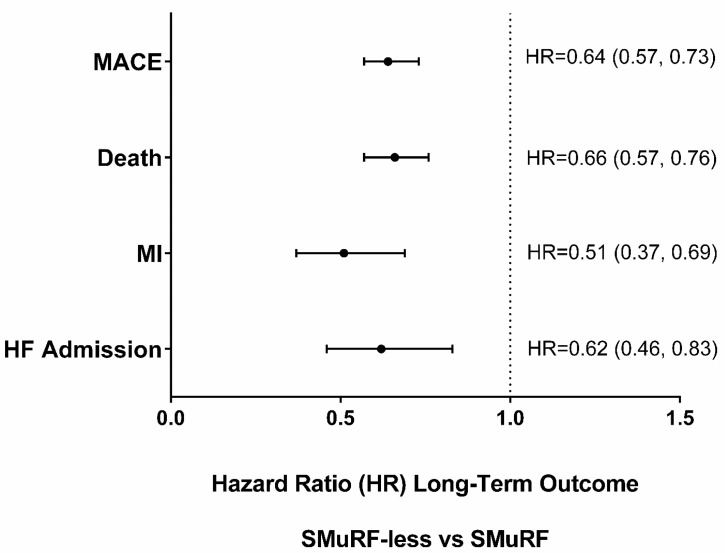
Long-term Outcomes adjusted Hazard Ratios (HRs) in SMuRF-less vs. SMuRF (referent) NSTEMI Patients.

**Figure 2:**
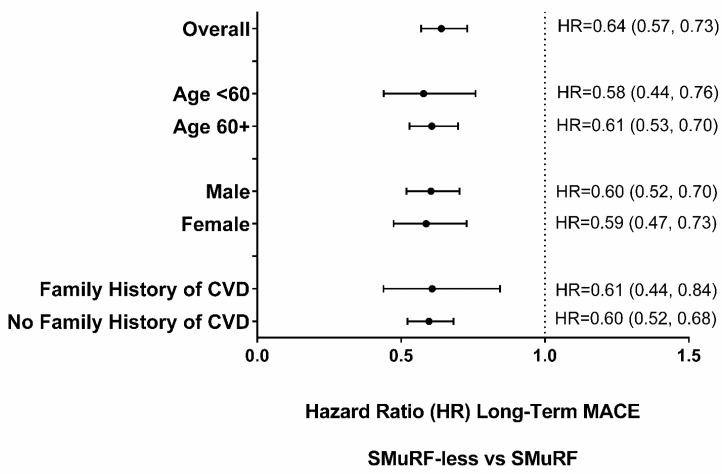
Long-term MACE adjusted Hazard Ratios (HR) for Subgroups by SMuRF Status.

One-year outcomes strongly favored SMuRF-less patients (Table 3). Long-term patients were followed for a mean of 4.8 years, median of 2.8, and range of 0–20.0, with long-term outcomes being less frequent in SMuRF-less patients (Table 3 and Figure 1). Results for key subgroups are shown in Figure 2. Total MACE was 19.6% vs. 39.3% in SMuRF-less vs. SMuRF patients, and each individual MACE category showed fewer events over the long-term in SMuRF-less patients, including all-cause death (16.0% vs. 33.1%), MI (2.95% vs. 7.0%), and HFH (3.5% vs. 7.6%) (all *p* < 0.001). These differences persisted when adjusted by baseline characteristics for total MACE (adj HR 0.64 (0.57, 0.73); *p* < 0.0001), death (adj HR 0.66 (0.57, 0.76); *p* < 0.0001), MI (adj HR 0.51 (0.37, 0.69); *p* < 0.0001), and HF hospitalization (adj HR 0.62 (0.46, 0.83); *p* = 0.001).

### 3.5. Subgroup Analyses

Long-term MACE by SMuRF status was examined for several patient subgroups (Figure 2). For all these subgroups, SMuRF-less patients were less likely to have long-term MACE.

We also examined outcomes by the number of SMuRF factors. Demographics and treatments did differ by the number of SMuRF factors (Supplementary Tables S1 and S2). When adjusted for these baseline differences, higher adjusted rates of MACE and death in the short-term and MACE, death, MI, and HFH long-term were found (Table 4). Each increase in SMuRF count was associated with an increase in the long-term events (adj HR 1.20 for both MACE and death, 1.29 for MI, and 1.22 for HF, all *p* < 0.0001) (Table 4). Similarly, when the counts of the SMuRF risk factors were examined categorically and compared to no (0) factors (SMuRF-less), there was an increased risk of outcomes with greater numbers of risk factors (3 or 4 vs. 1 or 2) (Supplementary Table S3). If we included former smokers in the no SMuRF category, outcome comparisons did not change (Supplementary Table S4).

## 4. Discussion

### 4.1. Summary of Study Findings

Our study observed the following key findings: first, the prevalence of SMuRF-less NSTEMI is relatively high—almost one-fifth of patients in our Intermountain experience. Second, the short-term (60-day) rates of MACE and death are lower than those of NSTEMI patients with SMuRF. Third, long-term outcomes also strongly favor SMuRF-less patients, with lower rates of total MACE, death, MI, and HF hospitalization, and these differences persisted after adjustment. The findings also were consistent through multiple subgroup analyses. A slightly higher rate of normal angiograms could be a contributing factor to a better outcome.

While recognizing deficiencies in SMuRF-less patients who are at greater risk for ACS, our findings indicate that in the current US practice combining interventional and medical therapy, favorable short- and long-term outcomes can be achieved for SMuRF-less compared to SMuRF patients with NSTEMI.

### 4.2. Literature Comparisons

Over the past 5 years, a number of reports from Australia, Europe, and Asia have assessed the prevalence and prognosis of patients without standard modifiable risk factors presenting with STEMI [1,2,3,4]. These studies have reported that these SMuRF-less STEMI patients are frequent (14–27% of presentations) and generally, although not universally, have shown a worse short-term prognosis [6]. Our recent study did confirm the high frequency of SMuRF-less STEMI presentations (26%) [6]. However, unlike several non-United States reports, adjusted early (<60 days) event rates were not higher in SMuRF-less STEMI patients, and long-term outcomes were favorable, with a reduced adjusted rate of total MACE and heart-failure hospitalizations. Moreover, high early mortality rates in non-United States studies were attenuated after adjustment for the reduced use of guideline-indicated treatments during the immediate post-infarction period [5].

NSTEMI presentations without standard modifiable risk factors only recently have received attention [3,7,8]. SMuRF-less NSTEMI was also reported to be common in these publications (7–22%), although generally less common than with STEMI presentations (14–27%). However, NSTEMI presentations are 2–3 times more frequent than STEMI presentations [3,6] and carry a higher long-term mortality risk [9], emphasizing their importance.

In a UK nationwide cohort study, more than one-fifth of patients presenting with NSTEMI had no SMuRFs [7]. These patients were less likely to receive guideline-directly medications. However, after propensity matching, these SMuRF-less NSTEMI patients were less likely to have short-term (all-cause and cardiac) mortality and total MACE (OR’s 0.85) compared to patients with SMuRFs. Our United States findings are consistent with these, both in terms of frequency and prognosis, and they extend to long-term relative outcomes.

Figtree et al. published results for SMuRF-less NSTEMI patients from the SWEDEHEART Registry [8]. A total of 11.2% of NSTEMI patients presented without modifiable risk factors. These patients had higher rates of all-cause (HR 1.20 (CI 1.10–1.30), *p* < 0.0001) and cardiovascular mortality at 30 days (HR 1.25 (CI 1.13–1.38)), and these differences remained after adjustment for age and sex. SMuRF-less patients received lower rates of statins, ACEI/ARBs, and beta-blockers. However, the long-term prognosis favored SMuRF-less patients who survived to 30 days.

In a recent study from Asia, SMuRF-less patients presenting with STEMI had an increased short-term risk of mortality. However, there were no differences in mortality in SMuRF-less versus SMuRF patients presenting with NSTEMI [3]. Differences compared to our study likely relate to different populations and treatment and analytical approaches. However, none of the NSTEMI studies have reported increased mortality, in contrast to the SMuRF-less STEMI literature.

### 4.3. Mechanistic Considerations and Non-Standard Risk Factors

Given that an important proportion of both NSTEMI and STEMI patients are SMuRF-less on presentation raises the question of unidentified risk factors. The international INTERHEART case-control study of myocardial infarction identified dietary patterns, physical activity, alcohol consumption, waist/hip ratio, and psychosocial factors to standard risk factors and achieved a 90–94% prediction of population-attributable risk [10].

Another important and increasingly recognized environmental risk factor not accounted for in standard risk assessment is air pollution. We and others have documented its important impact on MI incidence, especially in those with pre-existing diseases [11].

Both lipoprotein(a) and apolipoprotein-B (apoB) are new lipid-related factors that may improve risk stratification as well as target treatment [12,13,14,15].

Novel biomarkers represent another potential avenue to advance risk factor prediction. In one study of 30 novel biomarkers [16], a biomarker score including N-terminal pro-brain natriuretic peptide, C-reactive protein, and troponin-I improved the 10-year MACE estimate when added to a conventional risk model. In contrast, an earlier biomarker study did not find the incremental value of any of the 19 biomarkers tested [17]. Coagulation factor deficiencies also could play a role in SMuRF-less patients and would be deserving of future study.

Increasingly, whole exome and whole genome genetic analysis are possible, and polygenetic risk scores are being developed and tested for CAD prediction. In a whole-genome study in over 2000 multi-ethnic patients with early-onset MI, both monogenetic familial hypercholesterolemia mutations and a high polygenic risk score predicted an increased odds of early-onset MI (i.e., by more than 3-fold), but a high polygenetic risk score was 10-fold more common [18].

More recently, a polygenetic risk score for CAD (designated metaGRS) was developed by the UK Biobank using 1.6 million genetic variants. Patients falling into the top 20% of metaGRS had a hazard ratio of 4.2 compared to the bottom 20%, and metGRS was more predictive for incident CAD than conventional risk factors [19].

Imaging has the potential advantage of assessing actual disease presence as opposed to only disease probability. Of current imaging modalities, coronary artery calcium (CAC) scoring using computed tomography has emerged as the leading method [20]. Silverman et al. assessed CAC values at the extremes of traditional risk factor scores in the multi-ethnic Study of Atherosclerosis (MESA) [21]. At the extremes of these traditional risk factors, CAC distribution was heterogeneous. A high CAC burden among individuals without risk factors was associated with an elevated event rate, and an absent or mild CAC burden in those with a high-risk ractor burden had a lower-than-predicted event rate.

### 4.4. Clinical Implications

Our study, together with concurrent experience by others, emphasizes the importance of recognizing patients at risk for and presenting with NSTEMI, as well as with STEMI, but who do not have any of the four standard modifiable risk factors. Primary CV risk algorithms require improvement. Additional risk factors now need to be considered for optimal assessment of primary coronary risk. As noted above, some considerations include other lifestyle and environmental measures, newer lipids and, possibly, other biomarkers, and imaging tests (e.g., CAC). Genetic testing is currently reserved for those with suspected monogenetic disease (i.e., familial hyperlipidemia). However, in the near future, a polygenetic risk assessment may be available. SMuRF-less patients presenting with NSTEMI should not be considered to be at inherently low risk, and they should be managed aggressively with contemporary in-hospital and outpatient therapies. With this, their prognosis should be favorable.

### 4.5. Strengths and Limitations

A strength of this and its companion study [6] is its use of dedicated, longstanding, prospectively collected, and integrated catheterization laboratory and institutional electronic medical records systems. Another strength is a system-wide pathway for the management of acute coronary syndromes. A limitation is that of all retrospective, observational studies, in that, despite adjustment for multiple demographic factors, results are subject to uncorrected selection bias that may leave some selection bias unaccounted for. Prehospital events (e.g., out-of-hospital cardiac arrest/deaths) may not be completely ascertained. However, the consistency of our findings through multiple subgroup and sensitivity analyses has reassured us with respect to the unlikelihood of important residual selection bias. Our population is primarily of White/Caucasian racial/ethnic heritage; the degree of generalizability to other ethnic/racial groups is unclear. Results also may not apply to healthcare systems with substantially different approaches to ACS management. Some long-term events may have occurred outside of the Intermountain Healthcare system and be missed, although we doubt that this would favor one over another group by SMuRF status. Finally, the standard risk factors here and in the literature have been treated categorically, whereas they are continuous variables. To what extent sub-threshold values for blood pressure, lipids, and glycemia could differentiate SMuRF-less with and without incident myocardial infarction deserves investigation.

## 5. Conclusions

In our large United States healthcare population, NSTEMI presentation without standard modifiable risk factors was confirmed to be common, involving almost one-fifth of NSTEMI presentations. Consistent with other but not all reports, early (<60 days) event rates were lower in SMuRF-less NSTEMI patients, and, consistent with most reports, long-term outcomes were favorable, with a reduced adjusted rate of mortality, MI, and HF hospitalization. Further studies are needed to increase our understanding of non-traditional NSTEMI risk factors, prospective identification of SMuRF-less patients at increased ACS risk, and preventive and acute treatment implications for these SMuRF-less patients.

## Figures and Tables

**Table 1:** Baseline Characteristics of NSTEMI Patients by SMuRF Status.

	SMuRF		SMuRF-Less		
Demographics and Clinical Characteristics	*n* = 6738 (82.2%)		*n* = 1458 (17.8%)		*p*-Value
	*n*	%	*n*	%	
Age, median (IQR), years	67 (58, 76)		65 (54, 74)		<0.0001
Sex					0.04
Male	4533	67.28%	1021	70.03%	
Female	2205	32.72%	437	29.97%	
Race					0.02
White/Caucasian	6049	89.77%	1347	92.39%	
African American	51	0.76%	11	0.75%	
Asian	124	1.84%	21	1.44%	
Pacific Islander	16	0.24%	5	0.34%	
Unknown	498	7.39%	74	5.08%	
Family history of heart disease	2130	31.61%	170	11.66%	<0.0001
Comorbidities, No. (%)					
Atrial Fibrillation (AF)	1683	24.98%	285	19.55%	<0.0001
COPD	1127	16.73%	182	12.48%	<0.0001
Depression	1733	25.72%	306	20.99%	0.002
Heart Failure (HF)	819	12.15%	33	3.6%	<0.0001
Stroke	349	5.18%	41	2.81%	0.0001
SMuRF criteria					
Diabetes	3665	54.4%	0		NA
Hyperlipidemia	4948	73.4%	0		NA
Hypertension	4966	73.7%	0		NA
Smoking history					NA
Never	5235	77.7%	1458		
Former	823	12.2%	0		
Current	680	10.1%	0		

IQR = Interquartile Range. COPD = chronic obstructive pulmonary disease; Analyses: Chi-square (categorical) and Wilcoxon rank sum (continuous) tests were used to examine differences in baseline characteristics for those patients with and without SMuRF. SMuRF = standard modifiable risk factors.

**Table 2:** Interventions and Medications for NSTEMI Patients by SMuRF Status.

	SMuRF		SMuRF-Less		
Treatments and Medications	*n* = 2591		*n* = 919		
	*n*	%	*n*	%	*p*-Value
PCI performed	3195	58.1%	767	52.6%%	0.0001
CABG	869	12.9%	156	10.7%	0.02
Discharge Medications					
Beta Blocker	5685	84.4%	1226	84.1%	0.79
ACE-I/ARB	4153	61.6%	739	50.7%	<0.0001
Antiplatelet	6463	95.9%	1390	95.3%	0.31
Aspirin	6376	94.6%	1380	94.7%	0.97
Dual antiplatelet	3509	52.1%	807	55.4%	0.023
CCB	1490	22.1%	336	23.0%	0.44
Statin	6212	92.2%	1310	89.9%	0.0003

PCI = percutaneous coronary intervention; CABG = coronary artery bypass graft surgery; ACE-I = angiotensin-converting enzyme inhibitor; ARB = angiotensin receptor blocker; CCB = calcium channel blocker. Analyses: Chi-square tests were used to examine differences in treatments and medications for those patients with and without SMuRF.

**Table 3:** Outcomes of NSTEMI Patients by SMuRF Status.

	SMuRF		SMuRF-Less					
	*n* = 2591		*n* = 919					
	*n*	%	*n*	%	Unadjusted *p*-Values	Adj ^a^ HR	95% CI	*p*-Value
**60-day Outcomes**								
MACE	531	7.88%	59	4.05%	<0.0001	0.55	(0.42, 0.73)	<0.0001
Death	403	5.98%	49	3.36%	0.0001	0.62	(0.46, 0.84)	0.002
MI	47	0.70%	1	0.07%	0.02	NA ^b^		
HF Hospitalization	100	1.48%	11	0.75%	0.03	NA ^b^		
**1-year Outcomes**								
MACE	1019	15.12%	110	7.54%	<0.0001	0.56	(0.46, 0.69)	<0.0001
Death	725	10.76%	83	5.69%	<0.0001	0.62	(0.49, 0.78)	<0.0001
MI	178	2.64%	12	0.82%	0.0001	0.35	(0.19, 0.63)	0.0005
HF Hospitalization	214	3.18%	23	1.58%	0.0016	0.56	(0.36, 0.88)	0.011
**Long-term Outcomes**								
MACE	2647	39.28%	286	19.62%	<0.0001	0.64	(0.57, 0.73)	<0.0001
Death	2231	33.11%	233	15.98%	<0.0001	0.66	(0.57, 0.76)	<0.0001
MI	575	7.03%	43	2.95%	<0.0001	0.51	(0.37, 0.69)	<0.0001
HF Hospitalization	518	7.57%	51	3.50%	<0.0001	0.62	(0.46, 0.83)	0.0001

Analysis: Cox proportional hazard regression was used to examine outcomes adjusted for baseline differences comparing No SMuRF vs. SMuRF. ^a^ Adj= No SMuRF vs. SMuRF adjusted for age, gender, race (white vs. non-white), ACE/ARB, anti-coagulant, PCI, Family history of CVD, and prior history of AF, COPD, HF, stroke, and depression. ^b^ NA = no modeling done due to too few outcomes. ACE-I = angiotensin-converting enzyme inhibitors; AF = atrial fibrillation; COPD = chronic obstructive pulmonary disease; HF = heart failure; MACE = major adverse cardiovascular event; MI = myocardial infarction; HR = hazard ratio.

**Table 4:** Outcomes of NSTEMI Patients by SMuRF Count.

	SMuRF Count													
	0		1		2		3		4					
	*n* = 1458		*n* = 1650		*n* = 2230		*n* = 2460		*n* = 398					
	*n*	%	*n*	%	*n*	*n*	%	*n*	%	*n*	Unadjusted	Adj ** HR	95% CI	*p*-Value
											*p*-Values			
**60-day Outcomes**														
MACE	59	4.05%	130	7.88%	140	6.28%	225	9.15%	36	9.05%	<0.0001	1.17	(1.08, 1.26)	<0.0001
Death	49	3.36%	97	5.88%	107	4.80%	174	7.07%	25	6.28%	<0.0001	1.14	(1.05, 1.25)	0.0032
MI	1	0.07%	11	0.67%	13	0.58%	21	0.85%	2	0.50%	0.02	NA		
HF Hospitalization	11	0.75%	26	1.58%	22	0.99%	41	1.67%	11	2.76%	0.008	NA		
**1-year Outcomes**														
MACE	110	7.54%	228	13.82%	260	11.66%	452	18.37%	79	19.85%	<0.0001	1.21	(1.15, 1.28)	<0.0001
Death	83	5.69%	170	10.30%	189	8.48%	311	12.64%	55	13.82%	<0.0001	1.17	(1.10, 1.25)	<0.0001
MI	12	0.82%	34	2.06%	48	2.15%	85	3.46%	11	2.76%	<0.0001	1.35	(1.18, 1.55)	<0.0001
HF Hospitalization	23	1.58%	50	3.03%	48	2.15%	94	3.82%	22	5.53%	<0.0001	1.23	(1.08, 1.39)	0.0011
**Long-term Outcomes**														
MACE	286	19.62%	605	36.67%	743	33.32%	1074	43.66%	225	56.53%	<0.0001	1.20	(1.16, 1.24)	<0.0001
Death	233	15.98%	486	29.45%	644	28.88%	900	36.59%	201	50.50%	<0.0001	1.20	(1.16, 1.25)	<0.0001
MI	43	2.95%	103	6.24%	120	5.38%	210	8.54%	41	10.30%	<0.0001	1.29	(1.19, 1.40)	<0.0001
HF Hospitalization	51	3.50%	110	6.67%	133	5.96%	221	8.98%	54	13.57%	<0.0001	1.22	(1.13, 1.32)	<0.0001

Analysis: Cox proportional hazard regression was used to examine outcomes adjusted for baseline differences comparing each one count increase in SMuRF. Adj ** = Each one count increase in SMuRF adjusted for age, sex, ACE-I/ARB, CCB, AF, COPD, depression, HF, family history; NA = no modeling done due to too few outcomes. ACE-I = angiotensin-converting enzyme inhibitors; AF = atrial fibrillation; COPD = chronic obstructive pulmonary disease; HF = heart failure; MACE = major adverse cardiovascular event; MI = myocardial infarction; HR = hazard ratio.

## Data Availability

The data underlying this article cannot be shared publicly due to US privacy laws. Data are available upon reasonable request to the corresponding author.

## Supplementary Materials

The following supporting information can be downloaded at: https://www.mdpi.com/article/10.3390/jcm12093263/s1, Table S1: Baseline Characteristics of NSTEMI Patients by Count of SMuRF Factors; Table S2: Treatment and Medication of NSTEMI Patients by Count of SMuRF Factors; Table S3: Outcomes of NSTEMI Patients by SMuRF Count (with each count compared to no risk factor); Table S4: Outcomes of NSTEMI Patients by SMuRF Status With Former Smoking Not Included as a Risk Factor.
