# Supplementary material for: Frequency and Outcomes of Patients Presenting with Non-ST Elevation Myocardial Infarction (NSTEMI) without Standard Modifiable Risk Factors: A US Healthcare Experience
Source: J Clin Med. 2023 May 3;12(9):3263. doi: 10.3390/jcm12093263 (PMC10179010; doi:10.3390/jcm12093263)
Supplement: Supplementary file 1 [file jcm-12-03263-s001.zip › jcm-2311237-supplementary.pdf]

Supplementary Table S1: Baseline Characteristics of STEMI Patients by SMuRF Count

Demographics and Clinical Characteristics	SMuRF Count 0 n=919		1 n=834		2 n=1007		3 n=649		4 n=101		P-value
	n	%	n	%	n	%	n	%	n	%	
**Age, median (IQR)**	61 (52, 70)		60 (52, 69)		62 (54, 72)		62 (54, 71)		59 (52, 67)		0.09
**Age groups**											0.005
<40	49	5.3%	31	3.7%	31	3.1%	18	2.8%	5	5.0%	
40-49	140	15.2%	138	16.6%	128	12.7%	80	12.3%	14	13.9%	
50-59	228	24.8%	245	29.4%	276	27.4%	166	25.6%	33	32.7%	
60-69	271	29.5%	216	25.9%	269	26.7%	200	30.8%	32	31.7%	
70-79	150	16.3%	130	15.6%	199	19.8%	131	20.2%	11	10.9%	
>79	80	8.7%	74	8.9%	104	10.3%	54	8.3%	6	5.9%	
**Sex**											0.03
Male	709	77.2%	615	73.7%	744	73.9%	455	70.1%	71	70.3%	
Female	210	22.9%	219	26.5%	263	26.1%	194	29.9%	30	29.7%	
**Race**											0.03
White/Caucasian	818	89.0%	734	88.0%	899	89.3%	544	83.8%	83	82.2%	
African American	8	0.9%	5	0.6%	5	0.5%	4	0.6%	0	0.0%	
Asian	15	1.6%	19	2.3%	20	2.0%	18	2.8%	0	0.0%	
Pacific Islander	3	0.3%	2	0.2%	2	0.20%	1	0.2%	0	0.0%	
Unknown	75	8.2%	74	8.9%	81	8.0%	82	12.6%	18	17.8%	
**Family history of heart disease**	137	14.9%	237	28.4%	334	33.2%	221	34.1%	57	56.4%	<0.0001
**Comorbidities**											
Atrial Fibrillation (AF)	112	12.2%	119	14.3%	167	16.6%	121	18.6%	13	12.9%	0.005
COPD	52	5.7%	54	6.5%	91	9.0%	74	11.4%	15	14.9%	<0.001
Depression	136	14.8%	129	15.5%	153	15.2%	141	21.7%	15	14.9%	0.002
Heart Failure (HF)	33	3.6%	26	3.1%	48	4.8%	47	7.2%	5	5.0%	0.002
Stroke	12	1.3%	7	0.8%	16	1.6%	13	2.0%	1	1.0%	0.40
**SMURF Criteria**											
Diabetes	0	0%	291	34.9%	172	17.1%	480	74.0%	101	100%	NA
Hyperlipidemia	0	0%	136	16.3%	755	75.0%	621	95.7%	101	100%	NA
Hypertension	0	0%	182	21.8%	832	82.6%	621	95.7%	101	100%	NA
Smoking history											NA
Never	919	100%	609	73.0%	752	74.7%	424	65.3%	0	0%	

Former	0	0%	69	8.3%	93	9.2%	76	11.7%	47	46.5%
Current	0	0%	156	18.7%	162	16.1%	149	3.0%	54	53.5%

Supplementary Table S2: Interventions and Medications of STEMI Patients by SMuRF Count

Treatments and Medications	SMuRF Count 0 n=919		SMuRF Count 1 n=834		SMuRF Count 2 n=1007		SMuRF Count 3 n=649		SMuRF Count 4 n=101		P-value
	n	%	n	%	n	%	n	%	n	%	
PCI performed	778	84.7%	721	86.5%	897	89.1%	562	86.6%	87	86.1%	0.24
CABG	58	6.3%	55	6.6%	78	7.8%	65	10.0%	12	11.9%	0.02
**Discharge Medications**											
Beta Blocker	787	85.6%	699	83.8%	849	84.3%	555	85.5%	79	78.2%	0.31
ACE-I / ARB	576	62.7%	544	65.2%	678	67.3%	454	70.0%	63	62.4%	0.03
Anticoagulant	333	36.2%	280	33.6%	339	33.7%	231	35.6%	37	36.6%	0.68
Antiplatelet	887	95.7%	797	95.6%	968	96.1%	628	96.8%	97	96.0%	0.78
Aspirin	875	95.1%	787	94.4%	958	95.1%	620	95.5%	96	95.0%	0.88
Calcium channel blocker	100	10.9%	63	7.6%	126	12.5%	117	18.0%	8	7.9%	<0.0001

Supplementary Table S3: Outcomes of NSTEMI Patients by SMuRF Count (with each count compared to no risk factor)

	**Adj HR (95% CI); p-value**			
	**SMuRF 1 vs 0 count**	**SMuRF 2 vs 0 count**	**SMuRF 3 vs 0 count**	**SMuRF 4 vs 0 count**
**60-day Outcomes**				
MACE	1.94 (1.42, 2.64); <0.0001	1.40 (1.02, 1.90); 0.0354	1.97 (1.46, 2.65); <0.0001	2.29 (1.49, 3.52); 0.0002
Death	1.71 (1.21, 2.42); 0.0022	1.25 (0.88, 1.76); 0.2131	1.79 (1.29, 2.49); 0.0005	1.87 (1.14, 3.08); 0.0140
MI	NA	NA	NA	NA
HF Hospitalization	NA	NA	NA	NA
**1-year Outcomes**				
MACE	1.78 (1.42, 2.24); <0.0001	1.36 (1.08, 1.70); 0.0085	2.09 (1.68, 2.59); <0.0001	2.53 (1.87, 3.41); <0.0001
Death	1.70 (1.31, 2.22); <0.0001	1.24 (0.96, 1.62); 0.1035	1.83 (1.42, 2.35); <0.0001	2.28 (1.60, 3.25); <0.0001
MI	2.41 (1.25, 4.66); 0.009	2.55 (1.34, 4.83); 0.0042	3.86 (2.08, 7.16); <0.0001	3.17 (1.37, 7.32); 0.0069
HF Hospitalization	1.91 (1.61, 3.14); 0.0107	1.17 (0.71, 1.94); 0.5451	1.96 (1.22, 3.14); 0.0053	3.28 (1.79, 6.04); 0.0001
**Long-term Outcomes**				
MACE	1.47 (1.28, 1.69); <0.0001	1.28 (1.11, 1.47); 0.0006	1.87 (1.64, 2.14); <0.0001	2.20 (1.84, 2.63); <0.0001
Death	1.38 (1.18, 1.62); <0.0001	1.29 (1.11, 1.50); 0.0010	1.81 (1.56, 2.10); <0.0001	2.24 (1.84, 2.72); <0.0001
MI	1.73 (1.21, 2.47); 0.0027	1.58 (1.11, 2.25); 0.0114	2.71 (1.94, 3.79); <0.0001	2.64 (1.70, 4.09); <0.0001
HF Hospitalization	1.54 (1.10, 2.15); 0.110	1.25 (0.90, 1.73); 0.1902	1.91 (1.40, 2.61); <0.0001	2.60 (1.75, 3.86); <0.0001

Supplementary Table S4: Outcomes of STEMI Patients by SMuRF Status with Former Smoking Not Included as a Risk Factor

	SMuRF n=2522		No SMuRF n=988					
	n	%	n	%	Unadjusted p-values	Adj** HR	95% CI	p-value
**60-day Outcomes**								
MACE	207	8.2%	74	7.5%	0.52	0.93	(0.71, 1.21)	0.58
Death	168	6.7%	67	6.8%	0.87	1.02	(0.77, 1.36)	0.88
MI	14	0.6%	4	0.4%	0.58	NA*		
HF Hospitalization	26	1.0%	3	0.3%	0.04	NA*		
**Long-term Outcomes**								
MACE	795	31.5%	215	21.8%	0.002	0.81	(0.69, 0.94)	0.005
Death	631	25.0%	175	17.7%	0.02	0.84	(0.71, 1.00)	0.05
MI	175	6.9%	45	4.6%	0.11	0.80	(0.57, 1.11)	0.18
HF Hospitalization	123	4.9%	15	1.5%	0.0001	0.37	(0.21, 0.63)	0.0003

Analysis: Cox proportional hazard regression was used to examine outcomes adjusted for baseline differences comparing No SMuRF vs SMuRF.

Adj**= No SMuRF vs SMuRF adjusted for age, sex, ACE-I / ARB, AF, COPD, family history
*NA = no modeling done due to too few outcomes.

ACE-I = angiotensin converting enzyme inhibitors; AF = atrial fibrillation; COPD = chronic obstructive pulmonary disease; HF = heart failure; MACE = major adverse cardiovascular event; MI = myocardial infarction; HR=hazard ratio
